# Variability in amount of weight-bearing while performing weight-bearing radiographs for assessing stability of ankle fractures

**DOI:** 10.1007/s00068-024-02474-2

**Published:** 2024-03-02

**Authors:** Inge Zonneveld, Jochem Hoogendoorn

**Affiliations:** Haaglanden Medical Center (Trauma Unit), The Hague, The Netherlands

**Keywords:** Weight-bearing, Radiography, Type B fibula fracture, Medial clear space

## Abstract

**Purpose:**

Weight-bearing (WB) radiographs are commonly used to judge stability of type B fibula fractures and guide the choice of treatment. Stable fractures can be treated conservatively, and unstable fractures surgically. The question is raised how much weight patients actually put on their broken ankle while making a WB radiograph. The current study will give insight in the actual amount of WB in WB radiographs.

**Methods:**

In this retrospective cohort study, 57 patients with a type B fibula fracture with a medial clear space (MCS) < 6 mm on regular mortise (RM) view who underwent a WB radiograph were included.

We designed a ramp with a scale in the plateau where the radiographs were taken. Total body weight (TBW) and amount of WB on the fractured limb were measured.

**Results:**

The mean WB on the fractured limb was 49 (13–110) kg and the mean TBW was 79 (45–128) kg, calculating a mean percentage of WB of 63. The mean MCS on the RM radiograph was 3.0 mm, compared to 2.9 mm on the WB radiograph. The mean superior clear space (SCS) was 3.2 mm on the RM view, compared to 3.2 mm on the WB radiograph as well. The average fibular dislocation was 1.5 mm on the RM radiograph, compared to 1.6 mm on the WB radiograph.

**Conclusion:**

There is a big variability in the amount of weight-bearing on the ankle when a WB radiograph is made. This is important to keep in mind when assessing the radiographs and deciding on the treatment course.

## Introduction

Ankle fractures account for 8.2% of all fractures in the Netherlands [1]. The most common type is the trans-syndesmotic type B fibula fracture, which is commonly caused by a supination-external rotation (SER) injury [2, 3]. In isolated type B fibula fractures, the assessment of stability is fundamental in deciding the course of treatment [4]. The deep deltoid ligament (DDL) and the medial malleolus are considered the main stabilizers of the ankle during weight-bearing [5]. When an additional DDL rupture or medial malleolar fracture occurs in combination with an isolated distal fibula fracture, the fracture is considered unstable and therefore requires operative treatment [6]. Stable type B fibula fractures are mainly treated non-operatively with excellent outcomes [7]. Therefore, it is important to determine stability.

Weight-bearing (WB) radiographs can be used to detect instability of the ankle joint [4]. When full WB is applied, rupture of the DLL will give lateral displacement of the ankle joint in combination with external rotation of the talus and therefore widening of MCS [4]. The current existing literature on WB radiographs describes “full” or “equal” WB while performing the radiograph, but none of the current literature describes the actual amount of WB. An underlying assumption is that patients are placing 50% of their body weight on the fractured limb during WB radiographs [8]. But it is likely that when a patient is asked to put weight on their broken ankle, they will not put full weight on it due to discomfort.

This raises the question of how much weight is actually put on the broken ankle when a WB radiograph is made and if this affects the accuracy of the WB radiograph.

The purpose of this current prospective cohort study is to give an insight in the variability of the amount of WB on the ankle when a WB radiograph is made to define stability for isolated type B fibula fractures.

## Patients and methods

This study is a prospective single-center cohort study executed in the Netherlands. All patients were included who presented between September 2021 and July 2022 at the Emergency Department due to an isolated type B fibula fracture with a MCS < 6 mm on regular RM radiograph and who received an additional WB radiograph in which the total body weight (TBW) and WB weight were noted. Children under the age of 15 were excluded, and also patients with a MCS ≥ 6 mm on RM were excluded, because these injuries were considered unstable with an evident indication for surgical treatment.

**Fig. 1:**
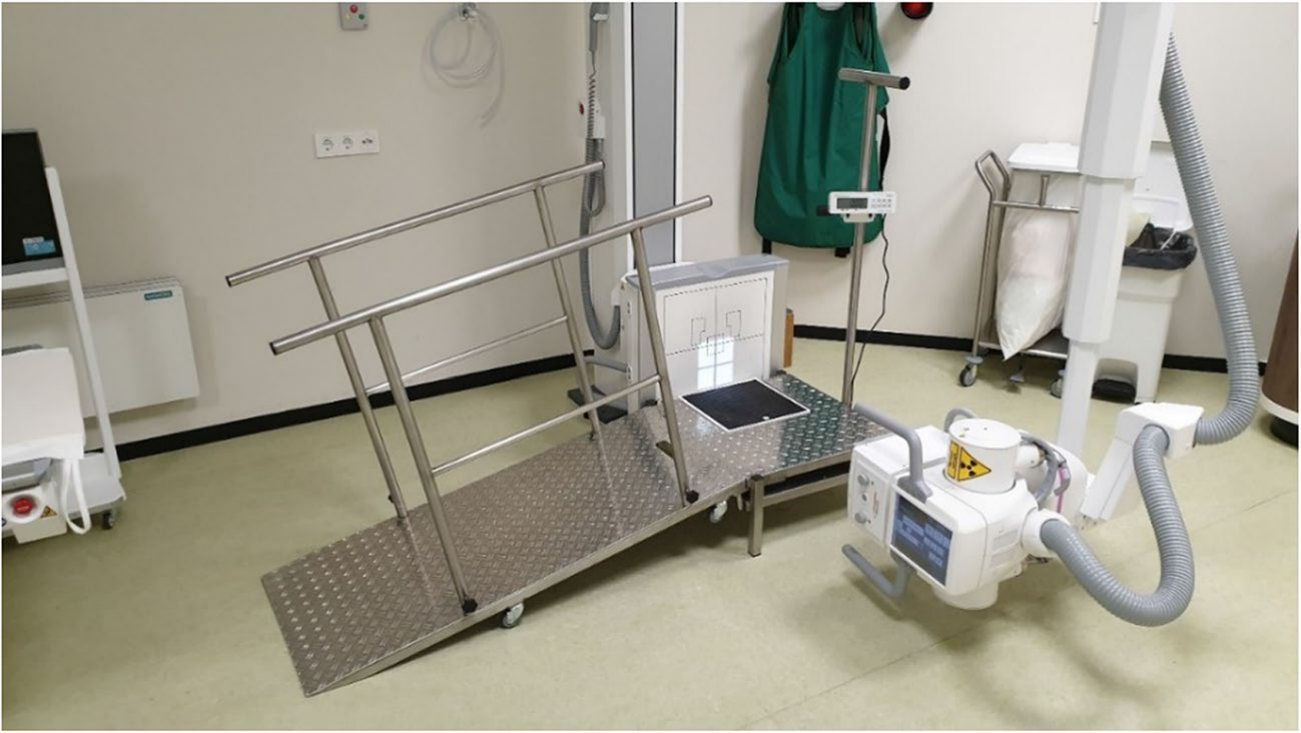
Weight-bearing measurement setup, tailor-made ramp leading up to the platform with build in scale

After the implementation of using WB radiographs to detect stability of ankle fractures in our clinic, we received feedback from the radiology technicians that patients were usually not able to step up to the plateau and often had to lean on the technician. Therefore, we designed a tailor-made ramp with handrails (Fig. 1).

**Fig. 2:**
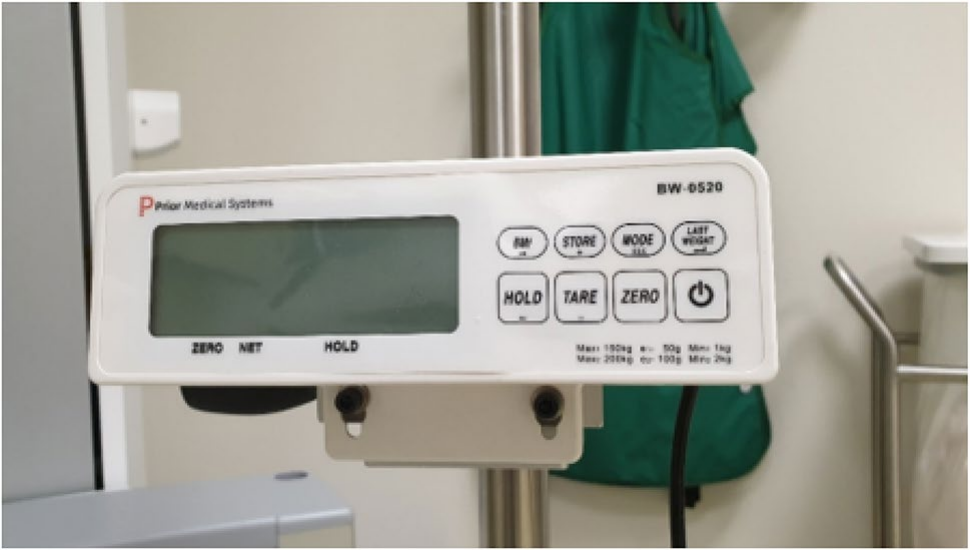
Scale display

**Fig. 3:**
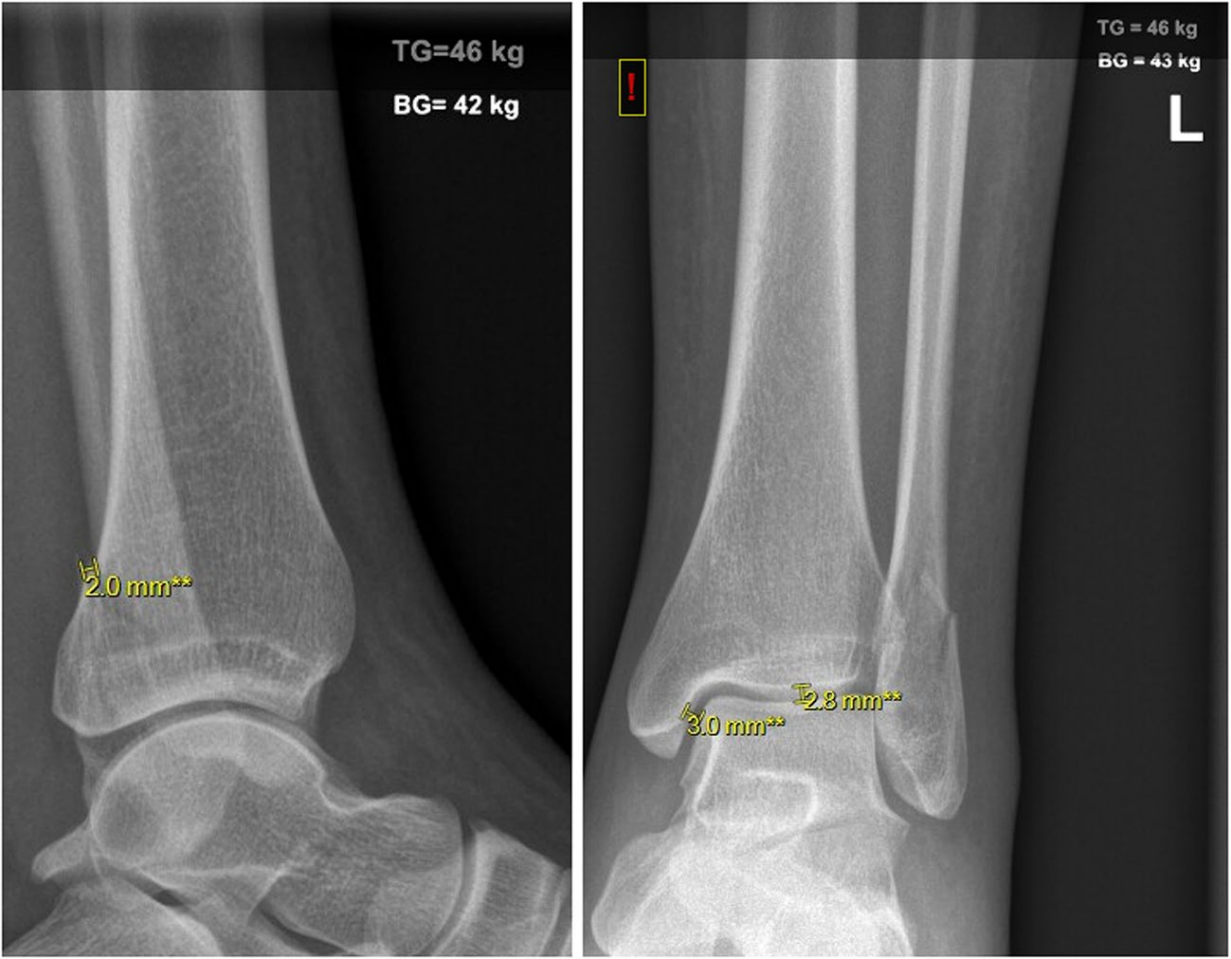
Weight-bearing mortise and lateral view radiographs of a 32-year-old female, 7 days after type B fibula fracture with a medial clear space (MCS) of 3.0 mm, superior clear space (SCS) of 2.8 mm, and fibular dislocation (FD) of 2.0 mm. TG, total body weight; BG, weight-bearing weight

This gave us the opportunity to build a digital scale into the plateau on which the radiographs were taken. Standing at the plateau, the patient was first asked to stand on the scale with both feet, after which the TBW was displayed at the scale display (Fig. 2). Then, the patient was asked to only place the fractured limb on the scale, with the heel against the detector, and to put as much weight as possible on the fractured limb while making the WB radiographs. Both a mortise and lateral views were made. In both radiographs, the TBW and the WB weight were noted (Fig. 3).

For each included patient, the following measurements were retrieved from the RM and the WB radiograph; MCS, SCS, and fibular dislocation. A digital calibrated ruler in Zillion PACS viewer P1 was used to measure the MCS and the SCS. The MCS was measured by drawing a line from the medial border of the talus to the lateral border of the medial malleolus. The SCS was measured between the distal tibia and the highest point of the talar dome. The fibular dislocation was measured on the lateral view radiograph. Examples of the measurements are shown in Fig. 3. All the measurements were taken on three separate moments; afterwards, the data was pooled and the mean was calculated. The calculated mean measurements of the MSC, SCS, and the fibular dislocation were used in the results.

The STROBE cohort checklist was used when writing this report [9]. The association between the percentage of weight-bearing and the difference in MCS between the RM and WB radiographs was calculated with a Pearson correlation coefficient. All statistical analyses were performed by using IBM Statistical Package for the Social Sciences (SPSS) version 27.0 [10]. Significance was set at the 5% level. GraphPad Prism version 9.0 was used for creating the scatter plots. Approval of the Regional Medical Ethics Committee was obtained (study number 17–134).

## Results

**Fig. 4:**
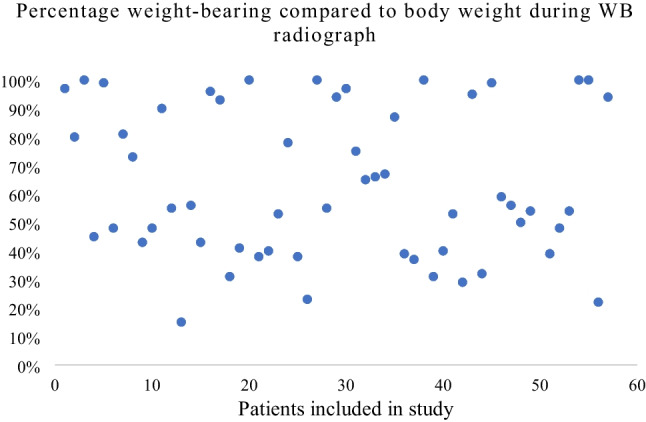
Scatter plot displaying the percentage of weight-bearing compared to body weight of each included patient

A total of 57 patients were included in this study, 27 males (47%) and 30 females (53%). The average age was 49.5 years (range 15–87, SD 20.5) at the time of injury. On average, the WB radiograph was made 5.7 days (range 0–21) after the first RM view. The mean TBW of the included patients was 79 kg (range 45–128) with a mean weight-bearing on the fractured limb of 49.3 kg (range 13–110). This calculates a mean percentage of weight-bearing of 63% (range 15–100). The results are displayed in Fig. 4.

**Fig. 5:**
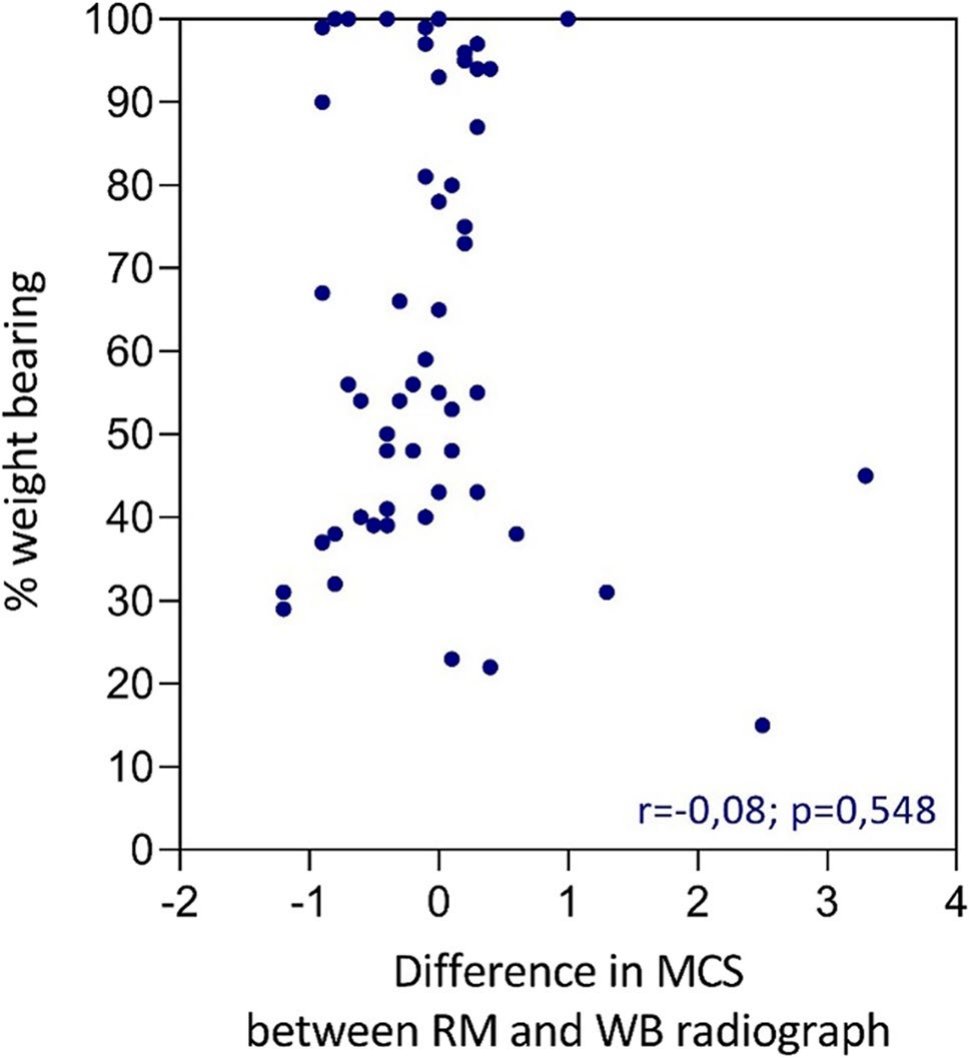
Scatter plot of correlation between % weight-bearing and difference in MCS between RM and WB radiograph in mm

The mean MCS on the RM radiograph was 3.0 ± 0.68 mm (1.6–4.3), compared to 2.9 ± 0.72 mm (1.6–6.0) on the WB radiograph. The Pearson correlation coefficient of the percentage of weight-bearing in relation to the difference in MCS between the RM and the WB radiographs was − 0.082 (*p* = 0.548), which concludes that there is no association between the percentage of weight-bearing and difference in MCS between the RM and the WB radiographs. The results are displayed in a scatter plot (Fig. 5).

The mean SCS was 3.2 ± 0.37 mm (2.4–4.1) on the RM view, while on the WB radiograph, the mean SCS widening was 3.2 ± 0.41 mm (2.4–4.1).

The average fibular dislocation was 1.5 ± 0.76 mm (0.0–3.9) on the RM radiograph, compared to 1.6 ± 0.76 mm (0.0–4.1) on the WB radiograph.

Fifty-six of 57 patients were considered to have a stable type B fibula fracture on the WB radiograph (MCS < 6.0 mm) and therefore treated non-operatively. The type of non-operative treatment varied between these patients. In most cases, at the ED, a plaster cast was applied, and if considered stable at the WB radiograph, the cast was replaced by a brace with advice for weight-bearing within pain limits for five more weeks. Some patients were treated with a soft cast for the total of 6 weeks, mainly due to higher age, overall health condition, and/or patient preferences. One patient, with a MCS of 3.5 mm on the RM radiograph, showed a MCS of 6.0 mm on the WB radiograph and was treated operatively with plate and screw fixation. No secondary dislocation or other complications occurred within our study group during the treatment time and follow-up period.

## Discussion

Isolated type B fibula fractures are a common type of injury. The course of treatment is based on the stability of the fracture.

It is common practice to determine incongruency on a regular ankle X-ray. In the national protocol of the Dutch Trauma Society and the Dutch guideline on ankle fractures, a medial clear space (MCS) ≥ 6 mm or MCS ≥ superior clear space (SCS) + 2 mm at regular mortise (RM) radiograph is defined to be an unstable injury in need for surgical treatment [4, 11].

However, around 54% of isolated type B fibular fractures are “borderline stable,” which means MCS between SCS and SCS + 2 mm [4, 6].

In current practice, around 40% of these borderline stable fractures are treated operatively [4, 7, 12–14]. An operation ensures direct anatomical reduction and prevents further displacement, but also comes with risks. A recent randomized controlled trial (*n* = 160 in 22 hospitals in Australia) concluded that surgery is not superior to non-surgical management for stable type B ankle fractures after 1 year and is associated with increased hospital admissions and adverse events [15]. Therefore, it is important to better determine stability.

WB radiographs can be used as a reliable diagnostic tool in type B fibula fractures [4, 16–18].

A recent prospective cohort study from Van Leeuwen et al. investigated the additional value of WB and gravity stress (GS) ankle radiographs to determine the stability of isolated type B ankle fractures [4].

In this study, a very high specificity of both GS and WB radiographs was found. With a cut-off value MCS ≥ SCS + 2 mm, the WB radiograph was 100% specific in excluding medial injury, with a sensitivity of 6% [4]. With this same cut-off value, the RM had a specificity of 97% and a sensitivity of 0% [4]. The GS has a sensitivity of 6% and specificity of 100% with cut-off value MCS ≥ SCS + 3 mm [4].

Holmes et al. and Seidel et al. also concluded that the GS radiograph has a higher MCS cut-off value compared to the mortise and WB radiograph and could, therefore, lead to a higher number of presumed instabilities and surgical overtreatment when using these same cut-off values of 4 or 5 mm [16, 18].

These studies support the hypothesis that WB radiographs identify fracture stability more closely to the actual clinical and biomechanical condition and that WB radiographs are appropriate to determine stability in isolated type B ankle fractures, with less unnecessary surgeries compared to GS radiographs [16].

When there is no widening of the MCS at the WB radiograph, the type B fibula fracture can be classified as stable and can be treated non-operatively with good functional outcomes [3, 16, 17].

Moreover, if fractures are considered stable after performing a WB radiograph, immediate full weight-bearing within pain limits in a brace can be advised. Previous literature of Van den Berg et al. comparing conservative treatment with a cast to functional treatment with a brace showed a significant difference at the 6-week follow-up for both pain score and range of motion in favor of the brace [19].

In our study, 41 of the 57 patients were treated with a brace. There was a large variability in the timing of switching to a brace. Before switching to a brace, the patient received a WB radiograph to determine stability during weight-bearing. Ten percent of the patients were able to receive a WB radiograph in the emergency department and were treated with a brace from the start, allowing immediate weight-bearing. A total of 62% of the patients switched from a cast to a brace somewhere between 3 days up to 3 weeks after injury, after a consultation at the outpatient clinic with a WB radiograph. For the patients not able to receive a WB radiograph in the emergency department due to pain, our recommendation would be to give a lower leg cast for 1 week and make the WB radiograph at the 1-week appointment at the outpatient clinic. When the fracture is considered stable at the WB radiograph, the patient can switch to a brace.

Unfortunately, not all patients were able to start weight-bearing because of some limiting factors (age, mobility, weight, or fear). Our study supports the findings of Van den Berg et al. that functional bracing is a safe and comfortable treatment option of stable type B fibula fractures for most patients.

The next question that arises is “what percentage of weight bearing is needed for a reliable WB radiograph?”. Currently, there is no evidence to suggest the recommended amount of weight that patients should put on the fractured limb to be able to adequately evaluate the WB radiographs. Although commonly not described, it is presumable that in previous studies, it was expected that the patient would put 50% of the total body weight on the fractured limb. Our present study however supports the hypothesis that not all patients are putting substantial weight on their fractured limb while making a WB radiograph. Figure 4 shows this great variability in the amount of weight-bearing, varying from 15 up to 100% of body weight. Twenty-two (39%) of the 57 patients were not able to put at least 50% of their body weight on the fractured limb. Therefore, we can conclude that the amount of weight-bearing in previous studies is presumably severely underestimated. The consequence of this finding is rather unknown.

To our knowledge, our study is the first cohort study that shows this large variability in the amount of weight-bearing in WB radiographs for type B fibula fractures.

Only one previous article, by Miller et al., has actually demonstrated and measured the amount of weight put on an injured joint while making a WB radiograph [8]. Each patient received two WB radiographs, the first without specific instruction on the amount of weight-bearing (trial 1) and the second with the specific instruction to place half [his or her] weight on the fractured limb (trial 2). They defined appropriate weight-bearing as > 45% of the TBW. The study showed substantial variability in percentage weight-bearing between patients (18–80%) [8]. In trial 1, 48% (24/50) of the patients were able to apply appropriate weight (> 45%) compared to 78% (39/50) in trial 2 (*p* = 0.002) [8]. Although specific instruction did make a significant difference, 22% (11/50) of the patients did not reach the appropriate amount of weight-bearing. However, this article included patients who required WB radiographs of the foot or ankle as part of their routine care, not describing the specific included injuries. It is likely that the amount of weight-bearing differs between different kinds of injuries of the foot and ankle, and therefore this study cannot be compared with our results.

A limitation of this study was the relatively small cohort. The explanation for this is that the tailor-made ramp with build-in scale is only present at one of the rooms at our radiology department. Therefore, not all the patients with a type B fibula fracture who presented for follow-up had their WB radiograph made at the tailor-made ramp with scale. For those patients, the TBW and WB weight were not noted in the WB radiographs, and these patients were therefore not included in this study.

In conclusion, this current study showed a large variability of weight applied in WB radiographs in patients with a type B fibula fracture. The historical assumption that patients will put 50% of their TBW on their fractured limb, when “equal weight bearing” WB radiographs are made, is presumably not correct. Our study shows that over one-third of the patients included did not meet this 50% weight-bearing, even when asked to put their TBW on the fractured limb.

Considering the above information, further studies are needed to determine the minimal amount of weight-bearing for a WB radiograph to be reliable.
